# Why Dilated Convolutional Neural Networks: A Proof of Their Optimality

**DOI:** 10.3390/e23060767

**Published:** 2021-06-18

**Authors:** Jonatan Contreras, Martine Ceberio, Vladik Kreinovich

**Affiliations:** Department of Computer Science, University of Texas at El Paso, El Paso, TX 79968, USA; jmcontreras2@utep.edu (J.C.); mceberio@utep.edu (M.C.)

**Keywords:** convolutional neural networks, dilated neural networks, optimality

## Abstract

One of the most effective image processing techniques is the use of convolutional neural networks that use convolutional layers. In each such layer, the value of the layer’s output signal at each point is a combination of the layer’s input signals corresponding to several neighboring points. To improve the accuracy, researchers have developed a version of this technique, in which only data from some of the neighboring points is processed. It turns out that the most efficient case—called dilated convolution—is when we select the neighboring points whose differences in both coordinates are divisible by some constant *ℓ*. In this paper, we explain this empirical efficiency by proving that for all reasonable optimality criteria, dilated convolution is indeed better than possible alternatives.

## 1. Introduction

### 1.1. Convolutional Layers: Input and Outpu

At present, one of the most efficient techniques in image processing and in other areas is a convolutional neural network; see, e.g., [1]. Convolutional neural networks include special types of layers that perform linear transformations.

Each such layer is characterized by integer-values parameters $\underline{X}\leq \bar{X}$, $\underline{Y}\leq \bar{Y}$, $d_{\mathrm{in}}\geq 1$, and $d_{\mathrm{out}}\geq 1$; then:

- the input to this layer consists of the values $F_{d^{\prime }}\left(x^{\prime },y^{\prime }\right)$, where $d^{\prime }$, $x^{\prime }$, and $y^{\prime }$ are integers for which $\underline{X}\leq x^{\prime }\leq \bar{X}$, $\underline{Y}\leq y^{\prime }\leq \bar{Y}$, and $1\leq d^{\prime }\leq d_{\mathrm{in}}$; and
- the output of this layer consists of the values $G_{d}\left(x,y\right)$, where *d*, *x*, and *y* are integers for which $\underline{X}\leq x\leq \bar{X}$, $\underline{Y}\leq y\leq \bar{Y}$, and $1\leq d\leq d_{\mathrm{out}}$.

### 1.2. Convolutional Layer: Transformation

A general linear transformation has the form

(1) $$G_{d}\left(x,y\right)=\sum_{d^{\prime }=1}^{d_{in}}\left(\sum_{x^{\prime }=\underline{X}}^{\bar{X}}\sum_{y^{\prime }=\underline{Y}}^{\bar{Y}}K_{d}\left(x,x^{\prime },y,y^{\prime },d^{\prime }\right)\cdot F_{d^{\prime }}\left(x^{\prime },y^{\prime }\right)\right),$$

for some coefficients $K_{d}\left(x,x^{\prime },y,y^{\prime },d^{\prime }\right)$.

Transformations performed by a convolutional layer are a specific case of such generic linear transformations, where the following two restrictions are imposed:

- first, each value $G_{d}\left(x,y\right)$ depends only on the values $F_{d^{\prime }}\left(x^{\prime },y^{\prime }\right)$, for which both differences $|x-x^{\prime }|$ and $|y-y^{\prime }|$ do not exceed some fixed integer *L*, and
- the coefficients $K_{d}\left(x,x^{\prime },y,y^{\prime },d^{\prime }\right)$ depend only on the differences $x-x^{\prime }$ and $y-y^{\prime }$:
  (2) $$K_{d}\left(x,x^{\prime },y,y^{\prime },d^{\prime }\right)=k_{d}\left(x-x^{\prime },y-y^{\prime },d^{\prime }\right)$$
  for some coefficients $k_{d}\left(i,j,d^{\prime }\right)$ defined for all pairs (i,j) for which |i|,|j|≤L.

The values $k_{d}\left(i,j,d^{\prime }\right)$ are known as a *filter*.

The resulting linear transformation takes the form

(3) $$G_{d}\left(x,y\right)=\sum_{d^{\prime }=1}^{d_{\mathrm{in}}}\left(\sum_{-L\leq i,j\leq L}k_{d}\left(i,j,d^{\prime }\right)\cdot F_{d^{\prime }}\left(x-i,y-j\right)\right).$$

**Figure 1:**
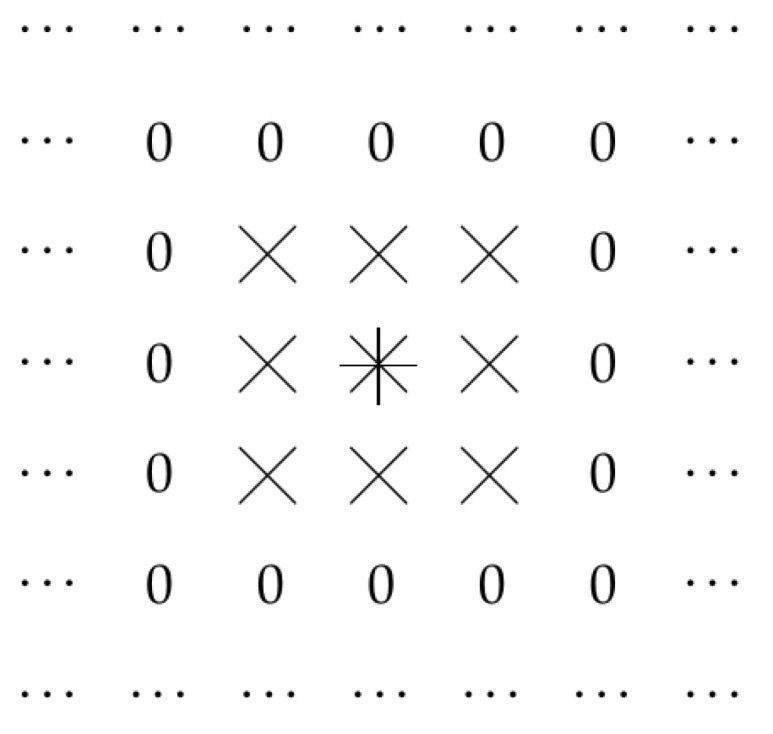
Convolution filter: case of L=1.

Thus, the output $G_{d}\left(x,y\right)$ of a convolutional layer corresponding to the point (x,y) is determined by the values $F_{d^{\prime }}\left(x-i,y-j\right)$ of the input to this layer at points (x−i,y−j) corresponding to |i|≤L and |j|≤L. This is illustrated by Figure 1, where, for L=1 and for a point (x,y) marked by an asterisk, we show all the points $\left(x^{\prime },y^{\prime }\right)=\left(x_{0}-i,y_{0}-j\right)$ that determine the values $G_{d}\left(x,y\right)$. For convenience, points $\left(x^{\prime },y^{\prime }\right)$ that do not affect the values $G_{d}\left(x,y\right)$, are marked by zeros.

**Figure 2:**
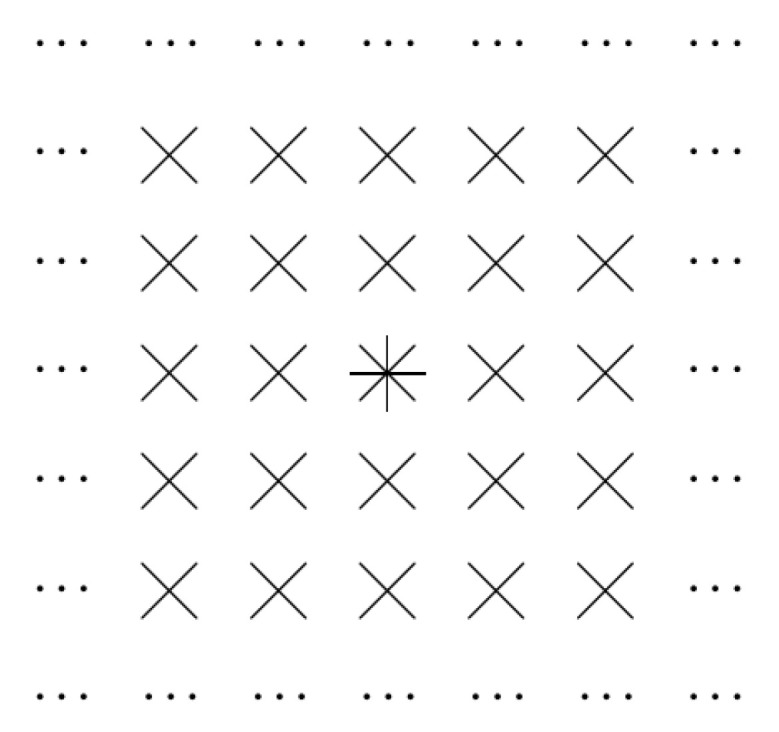
Convolution filter: case of L=2.

For L=2, a similar picture has the following form. This is illustrated by Figure 2.

### 1.3. Sparse Filters and Dilated Convolution

Originally, convolutional neural networks used filters in which all the values $k_{d}\left(i,j,d^{\prime }\right)$ for |i|,|j|≤L can be non-zero. It turned out, however, that we can achieve a better accuracy if we consider sparse filters, i.e., filters in which, for some pairs (i,j) with |i|,|j|≤L, all the values $k_{d}\left(i,j,d^{\prime }\right)$ are fixed at 0; see, e.g., [2,3,4].

**Figure 3:**
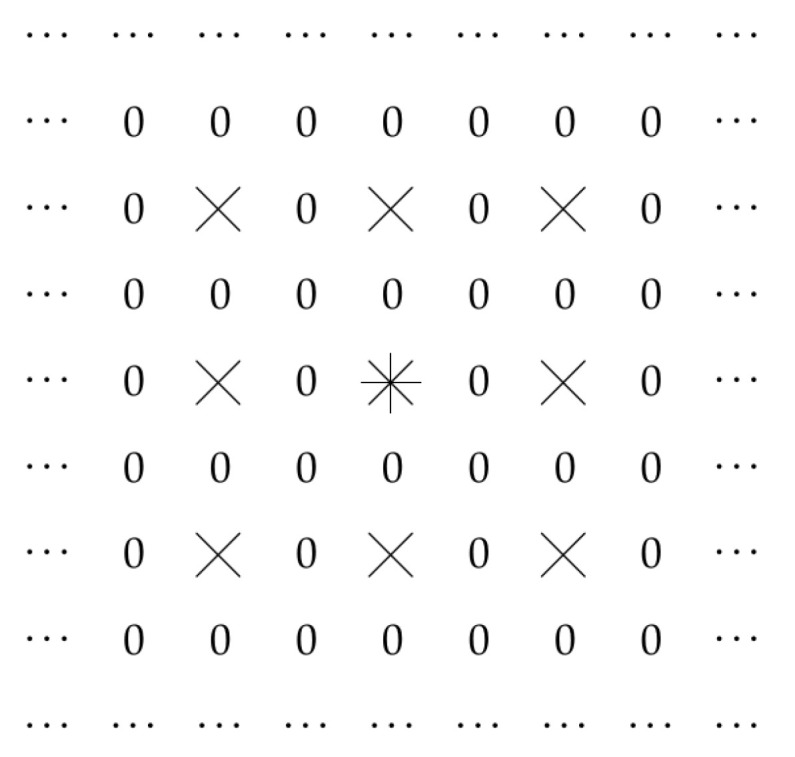
Case when L=2 and only values $k_{d}\left(i,j,d^{\prime }\right)$ with even *i* and *j* can be no-zero.

In Figure 3, we show an example of such a situation, when L=2 and only values $k_{d}\left(i,j,d^{\prime }\right)$, for which both *i* and *j* are even allowed to be non-zero.

In general, it turned out that such a restriction works best if we only allow $k_{d}\left(i,j,d^{\prime }\right)\neq 0$ for pairs (i,j) which are divisible by some integer *ℓ*, i.e., if we take

(4) $$G_{d}\left(x,y\right)=\sum_{d^{\prime }=1}^{d=d_{\mathrm{in}}}\left(\sum_{-L\leq i,j\leq L:\;i/\ell \in Z,\;j/\ell \in Z}k_{d}\left(i,j,d^{\prime }\right)\cdot F_{d^{\prime }}\left(x-i,y-j\right)\right).$$

In this case, the layer’s output signal $G_{d}\left(x,y\right)$ can be written in the following equivalent form:

(5) $$G_{d}\left(x,y\right)=\sum_{d^{\prime }=1}^{d_{\mathrm{in}}}\left(\sum_{-\tilde{L}\leq \tilde{i},\tilde{j}\leq \tilde{L}}{\tilde{k}}_{d}\left(\tilde{i},\tilde{j},d^{\prime }\right)\cdot F_{d^{\prime }}\left(x-\ell \cdot \tilde{i},y-\ell \cdot \tilde{j}\right)\right),$$

where we denoted $\tilde{L}\overset{\mathrm{def}}{=}L/\ell$, $\tilde{i}\overset{\mathrm{def}}{=}i/\ell$, $\tilde{j}\overset{\mathrm{def}}{=}j/\ell$, and ${\tilde{k}}_{d}\left(\tilde{i},\tilde{j},d^{\prime }\right)\overset{\mathrm{def}}{=}k\left(\ell \cdot \tilde{i},\ell \cdot \tilde{j},d^{\prime }\right)$.

The resulting networks are known as dilated convolutional neural networks, since skipping some points (i,j) in the description of the filter is kind of equivalent to extending (dilating) the distance between the remaining points; see, e.g., [2,3,4].

### 1.4. Empirical Fact That Needs Explanation

**Figure 4:**
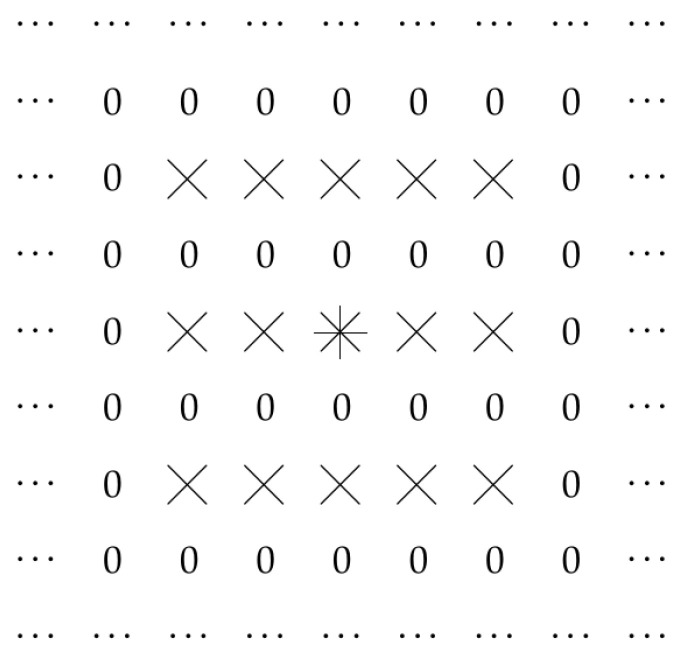
Case when L=2 and only values $k_{d}\left(i,j,d^{\prime }\right)$ with even *j* can be non-zero.

In principle, we could select other points (i,j) at which the filter can be non-zero. For example, we could select points for which *j* is even, but *i* can be any integer. This is illustrated by Figure 4.

**Figure 5:**
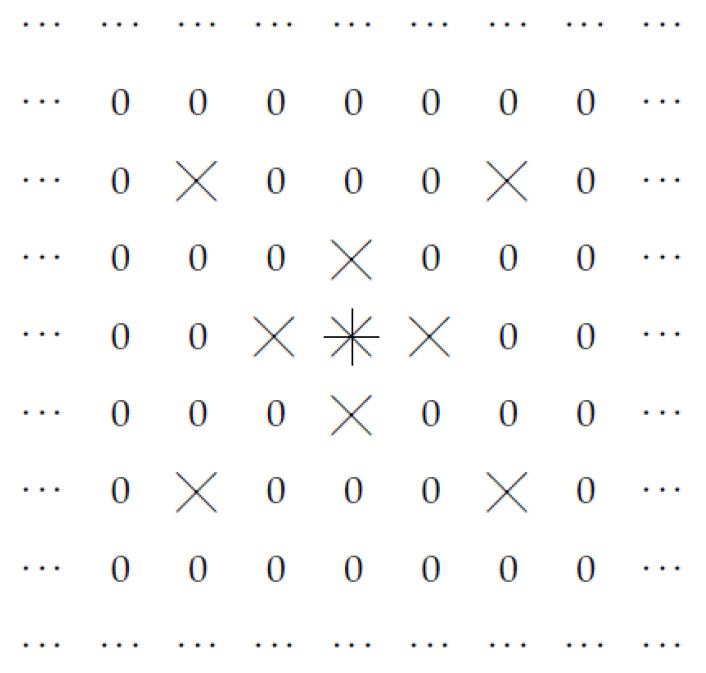
A possible selection of points (i,j) for which $k_{d}\left(i,j,d^{\prime }\right)$ can be no-zero.

Alternatively, for L=2, as points (i,j) at which $k_{d}\left(i,j,d^{\prime }\right)$ can be non-zero, we could select the points (0,0), (0,±1), (±1,0), and (±2,±2), see Figure 5.

However, empirical evidence shows that the selection corresponding to dilated convolution—when we select points for which *i* and *j* are both divisible by some integer *ℓ*—works the best [2,3,4].

To the best of our knowledge, there is no theoretical explanation for this empirical result—that dilated convolution leads to better results that select other sets of non-zero-valued points (i,j). The main objective of this paper is to provide such an explanation.

*Comment.* Let us emphasize that the only objective of this paper is to explain this empirical fact; we are not yet at a stage where we can propose a new method or even any improvements to the known methods.

## 2. Analysis of The Problem

### 2.1. Let Us Reformulate This Situation in Geometric Terms: Case of Traditional Convolution

In the original convolution Formula (1), to find the values $G_{d}\left(x,y\right)$, the layer’s output signal at a point (x,y), we need to know the values $F_{d^{\prime }}\left(x^{\prime },y^{\prime }\right)$, the layer’s input signal at all the points $\left(x^{\prime },y^{\prime }\right)$ of the type (x−i,y−j) for |i|,|j|≤L. We can reformulate it by saying that we need to know the values $F_{d^{\prime }}\left(x^{\prime },y^{\prime }\right)$ at all the points $\left(x^{\prime },y^{\prime }\right)$ at which the $\ell_{\infty }$ distance

(6) $$d_{\infty }\left(\left(x,y\right),\left(x^{\prime },y^{\prime }\right)\right)\overset{\mathrm{def}}{=}\max(|x-x^{\prime }|,|y-y^{\prime }\left|\right),$$

does not exceed *L*:

(7) $$G_{d}\left(x,y\right)=\sum_{d^{\prime }=1}^{d_{\mathrm{in}}}\left(\sum_{\left(x^{\prime },y^{\prime }\right)\in D:\;d_{\infty }\left(\left(x,y\right),\left(x^{\prime },y^{\prime }\right)\right)\leq L}k_{d}\left(x-x^{\prime },y-y^{\prime },d^{\prime }\right)\cdot F_{d^{\prime }}\left(x^{\prime },y^{\prime }\right)\right),$$

where we denoted

(8) $$D\overset{\mathrm{def}}{=}\left(Z\cap \left[\underline{X},\bar{X}\right]\right)\times \left(Z\cap \left[\underline{Y},\bar{Y}\right]\right).$$

We use, in this formula, the bounded subset *D* of the “grid” Z×Z and not the whole set $\tilde{S}\overset{\mathrm{def}}{=}Z\times Z$ only matters at the border of the domain *D*. So, to simplify our formulas, we can follow the usual tradition (see, e.g., [3]) and simply use the whole set $\tilde{S}=Z\times Z$ instead of the bounded set *D*:

(9) $$G_{d}\left(x,y\right)=\sum_{d^{\prime }=1}^{d_{\mathrm{in}}}\left(\sum_{\left(x^{\prime },y^{\prime }\right)\in \tilde{S}:\;d_{\infty }\left(\left(x,y\right),\left(x^{\prime },y^{\prime }\right)\right)\leq L}k_{d}\left(x-x^{\prime },y-y^{\prime },d^{\prime }\right)\cdot F_{d^{\prime }}\left(x^{\prime },y^{\prime }\right)\right).$$

*Comment.* Note that the set $\tilde{S}$ is potentially infinite. What makes the set of all the points $\left(x^{\prime },y^{\prime }\right)$—which affects the values $G_{d}\left(x,y\right)$—finite is the restriction $d_{\infty }\left(\left(x,y\right),\left(x^{\prime },y^{\prime }\right)\right)\leq L$, whose meaning is that such points $\left(x^{\prime },y^{\prime }\right)$ should belong to the corresponding neighborhood of the point (x,y).

### 2.2. Case of Dilated Convolution

The dilated convolution can be described in a similar way. Namely, we can describe the Formula (4) as

(10) $$G_{d}\left(x,y\right)=\sum_{d^{\prime }=1}^{d_{\mathrm{in}}}\left(\sum_{\left(x^{\prime },y^{\prime }\right)\in S_{\ell }\left(x,y\right):\;d_{\infty }\left(\left(x,y\right),\left(x^{\prime },y^{\prime }\right)\right)\leq L}k_{d}\left(x-x^{\prime },y-y^{\prime },d^{\prime }\right)\cdot F_{d^{\prime }}\left(x^{\prime },y^{\prime }\right)\right),$$

where $S_{\ell }\left(x,y\right)$ denotes the set of all the points $\left(x^{\prime },y^{\prime }\right)$ for which both differences $x-x^{\prime }$ and $y-y^{\prime }$ are divisible by *ℓ*:

(11) $$S_{\ell }\left(x,y\right)\overset{\mathrm{def}}{=}\left\{\left(x^{\prime },y^{\prime }\right):x^{\prime }\equiv x\mathrm{mod}\ell ,\;y^{\prime }\equiv y\mathrm{mod}\ell \right\}.$$

Note that, in this representation of dilated convolution, while we have several different sets $S_{\ell }\left(x,y\right)$ for different points (x,y), there is only one filter $k_{d}\left(x-x^{\prime },y-y^{\prime },d^{\prime }\right)$, namely the same filter that was used in the original representation (4). So, in this new representation, we have exactly as many parameters as before.

The main difference between this formula and the Formula (9) is that, in contrast to the usual convolution (9), where the same set $\tilde{S}=Z\times Z$ could be used for all the points (x,y), here, in general, we may need different sets $S_{\ell }\left(x,y\right)$ for different points (x,y).

For example, if ℓ=2, then we need four such sets:

- for points (x,y) for which both *x* and *y* are even, the Formula (10) holds for
  (12) $$S_{2}\left(0,0\right)=S_{2}\left(0,2\right)=\ldots =S_{0,0}^{\left(\ell =2\right)}\overset{\mathrm{def}}{=}\left\{\left(x,y\right)\in Z\times Z:x\mathrm{and}y\mathrm{are}\mathrm{even}\right\};$$
- for points (x,y) for which *x* is even but *y* is odd, the Formula (10) holds for

(13) $$S_{2}\left(0,1\right)=S_{2}\left(0,3\right)=\ldots =S_{0,1}^{\left(\ell =2\right)}\overset{\mathrm{def}}{=}\left\{\left(x,y\right)\in Z\times Z:x\mathrm{is}\mathrm{even}\mathrm{and}y\mathrm{is}\mathrm{odd}\right\};$$

- for points (x,y), for which *x* is odd but *y* is even, the Formula (10) holds for

(14) $$S_{2}\left(1,0\right)=S_{2}\left(1,2\right)=\ldots =S_{1,0}^{\left(\ell =2\right)}\overset{\mathrm{def}}{=}\left\{\left(x,y\right)\in Z\times Z:x\mathrm{is}\mathrm{odd}\mathrm{and}y\mathrm{is}\mathrm{even}\right\};$$

- finally, for points (x,y) for which *x* and *y* are both odd, the Formula (10) holds for
  (15) $$S_{2}\left(0,1\right)=S_{2}\left(0,3\right)=\ldots =S_{1,1}^{\left(\ell =2\right)}\overset{\mathrm{def}}{=}\left\{\left(x,y\right)\in Z\times Z:x\mathrm{and}y\mathrm{are}\mathrm{odd}\right\}.$$

In this case, instead of the single set $S_{1}\left(x,y\right)=\tilde{S}$ (as in the case of the traditional convolution), we have a set of such sets

(16) $$F=\left\{S_{0,0}^{\left(\ell =2\right)},S_{0,1}^{\left(\ell =2\right)},S_{1,0}^{\left(\ell =2\right)},S_{1,1}^{\left(\ell =2\right)}\right\}.$$

To avoid confusion, we will call subsets of the original “grid” Z×Z *sets*, while the set of such sets will be called a *family*. In these terms, the Formula (10) can be described as follows:

(17) $$G_{d}\left(x,y\right)=\;\sum_{d^{\prime }=1}^{d_{\mathrm{in}}}\left(\sum_{\left(x^{\prime },y^{\prime }\right)\in S_{F}\left(x,y\right):\;d_{\infty }\left(\left(x,y\right),\left(x^{\prime },y^{\prime }\right)\right)\leq L}k_{d}\left(x-x^{\prime },y-y^{\prime },d^{\prime }\right)\cdot F_{d^{\prime }}\left(x^{\prime },y^{\prime }\right)\right),$$

where $S_{F}\left(x,y\right)$ denotes the set S∈F from the family F that contains the point (x,y):

(18) $$\left(x,y\right)\in S_{F}\left(x,y\right)\mathrm{and}S_{F}\left(x,y\right)\in F.$$

In this representation, all four sets *S* from the family F are infinite—just like the set $\tilde{S}$ corresponding to the traditional convolution is infinite. Similarly to the traditional convolution, what makes the set of all the points $\left(x^{\prime },y^{\prime }\right)$—which affects the values $G_{r}\left(x,y\right)$—finite is the restriction $d_{\infty }\left(\left(x,y\right),\left(x^{\prime },y^{\prime }\right)\right)\leq L$, whose meaning is that such points $\left(x^{\prime },y^{\prime }\right)$ should belong to the corresponding neighborhood of the point (x,y).

**Figure 6:**
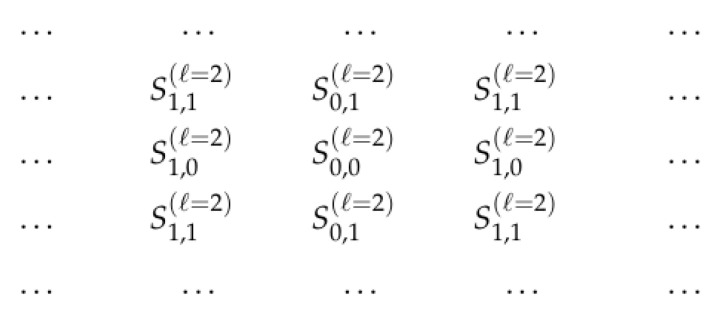
Sets $S_{F}\left(x,y\right)$ corresponding to different points (x,y)—for filters presented in Figure 3.

Figure 6 describes which of the four sets S∈F corresponds to each point (x,y) from the “grid” Z×Z:

For ℓ=3, we can get a similar reformulation, with the family

(19) $$F=\left\{S_{0,0}^{\left(\ell =3\right)},S_{0,1}^{\left(\ell =3\right)},S_{0,2}^{\left(\ell =3\right)},S_{1,0}^{\left(\ell =3\right)},S_{1,1}^{\left(\ell =3\right)},S_{1,2}^{\left(\ell =3\right)},S_{2,0}^{\left(\ell =3\right)},S_{2,1}^{\left(\ell =3\right)},S_{2,2}^{\left(\ell =3\right)}\right\},$$

where $S_{i,j}^{\left(\ell =3\right)}$ is the set of all the pairs (x,y)∈Z×Z, in which both differences x−i and y−j are divisible by 3.

In general, for an arbitrary point (x,y), we should use the set $S_{F}=S_{x\mathrm{mod}\ell ,\;y\mathrm{mod}\ell }^{\left(\ell =2\right)}.$

### 2.3. Other Cases

Such a representation is possible not only for dilated convolution. For example, the above case when we allow arbitrary value *i* and require the value *j* to be even can be described in a similar way, with

(20) $$F=\{S_{0},S_{1}\},$$

where:

- for points (x,y) for which *y* is even, we take
  (21) $$S_{F}\left(0,0\right)=S_{F}\left(1,0\right)=\ldots =S_{0}\overset{\mathrm{def}}{=}\left\{\left(x,y\right)\in Z\times Z:y\mathrm{is}\mathrm{even}\right\},$$
- and for points (x,y) for which *y* is odd, we take
  (22) $$S_{F}\left(0,1\right)=S_{F}\left(1,1\right)=\ldots =S_{1}\overset{\mathrm{def}}{=}\left\{\left(x,y\right)\in Z\times Z:y\mathrm{is}\mathrm{odd}\right\}.$$

We can also have families which have an infinite number of sets; an example of such a family will be given below.

We can also, in principle, consider the situations when we do not require that the coefficients $k_{d}\left(x,x^{\prime },y,y^{\prime },d^{\prime }\right)$ depend only on the differences $x-x^{\prime }$ and $y-y^{\prime }$. Thus, we arrive at the following general description.

### 2.4. General Case

In the general case, we get the following situation:

- we have a family F of subsets of the “grid” Z×Z;
- the values $G_{d}\left(x,y\right)$ of the layer’s output signal at a point (x,y) are determined by the formula
  (23) $$G_{d}\left(x,y\right)=\sum_{d^{\prime }=1}^{d_{\mathrm{in}}}\left(\sum_{\left(x^{\prime },y^{\prime }\right)\in S_{F}\left(x,y\right):\;d_{\infty }\left(\left(x,y\right),\left(x^{\prime },y^{\prime }\right)\right)\leq L}K_{d}\left(x,x^{\prime },y,y^{\prime },d^{\prime }\right)\cdot F_{d^{\prime }}\left(x^{\prime },y^{\prime }\right)\right),$$
  for some values $K_{d}\left(x,x^{\prime },y,y^{\prime },d^{\prime }\right)$, where $S_{F}\left(x,y\right)$ denotes the set S∈F from the family F that contains the point (x,y).

For the Formula (23) to uniquely determine the values $G_{d}\left(x,y\right)$, we need to make sure that the set $S_{F}\left(x,y\right)$ is uniquely determined by the point (x,y), i.e., that for each point (x,y), the family F contains one, and only one, set *S* that contains this point. In other words:

- different sets from the family F must be disjoint, and
- the union of all the sets S∈F must coincide with the whole “grid” Z×Z.

In mathematical terms, the family F must form a partition of the “grid” Z×Z.

*Comment.* To avoid possible confusion, it is worth mentioning that while *different sets* *S* from the family F are disjoint, this does not preclude the possibility that sets $S_{F}\left(x,y\right)$ and $S_{F}\left(x^{\prime },y^{\prime }\right)$ corresponding to *different points* $\left(x,y\right)\neq \left(x^{\prime },y^{\prime }\right)$ can be identical. For example, in the description of the traditional convolution, the family F consists of only one set $F=\left\{\tilde{S}\right\}$. In this case, for all points (x,y) and $\left(x^{\prime },y^{\prime }\right)$, we have $S_{F}\left(x,y\right)=S_{F}\left(x^{\prime },y^{\prime }\right)=\tilde{S}$.

In terms of sets corresponding to different points, disjointness means that *if* the sets $S_{F}\left(x,y\right)$ and $S_{F}\left(x^{\prime },y^{\prime }\right)$ are different, *then* these sets must be disjoint: $S_{F}\left(x,y\right)\cap S_{F}\left(x^{\prime },y^{\prime }\right)=\emptyset$.

### 2.5. We Do Not a Priori Require Shift-Invariance

Please note that we do not a priori require that the sets $S_{F}\left(x,y\right)$ and $S_{F}\left(x_{0},y_{0}\right)$ corresponding to two different points (x,y) and $\left(x_{0},y_{0}\right)$ should be obtained from each other by shift—this property is known as *shift invariance* and is satisfied both for the usual convolution and for the dilated convolution.

It should be emphasized, however, that we will show that this shift-invariance property holds for the optimal arrangement.

### 2.6. Let Us Avoid the Degenerate Case

From a purely mathematical viewpoint, we can have a partition of the “grid” Z×Z into one-point sets {(x,y)}. This is an example when the family F has infinitely many subsets.

In this case, no matter what value *L* we choose, the Formula (23) implies that the values $G_{d}\left(x,y\right)$ of the layer’s output signal at a point (x,y) are determined only by the values $F_{d^{\prime }}\left(x,y\right)$ of the layer’s input at this same point. This is equivalent to using a convolution with L=0; such a convolution is known as the 1-by-1 convolution.

While such a convolution is often useful, in this case, for each point (x,y), there is only one point $\left(x^{\prime },y^{\prime }\right)=\left(x,y\right)$, so it is not possible to select only some of the points $\left(x^{\prime },y^{\prime }\right)$—which is the whole idea of dilation. Since, in this paper, we study dilation, we will therefore avoid this 1-by-1 situation and additionally require that at least one set from the family F should contain more than one element.

### 2.7. What We Plan to Do

We will consider all possible families F that form a partition of the “grid” Z×Z, and we will show that for all optimality criteria that satisfy some reasonable conditions, the optimal family is either the family of sets corresponding to the dilated convolution—or a natural modification of this family.

Let us describe what we mean by optimality criteria.

### 2.8. What Does “Optimal” Mean?

In our case, we select between different families of sets F, $F^{\prime }$, …. In general, we select between alternatives *a*, *b*, etc. Out of all possible alternatives, we want to select an optimal one. What does “optimal” mean?

In many cases, “optimal” is easy to describe:

- we have an objective function f(a) that assigns a numerical value to each alternative *a*—e.g., the average approximation error of the numerical method *a* for solving a system of differential equations, and
- optimal means that we select an alternative for which the value of this objective function is the smallest possible (or, for some objective functions, the largest possible).

However, this is not the only possible way to describe optimality.

For example, if we are minimizing the average approximation error, and there are several different numerical methods with the exact same smallest value of average approximation error, then we can use this non-uniqueness to select, e.g., the method with the shortest average computation time. In this case, we have, in effect, a more complex preference relation between alternatives than in the case when decision is made based solely on the value of the objective function. Specifically, in this case, an alternative *b* is better than the alternative *a*—we will denote it by a<b—if:

- either we have f(b)<f(a),
- or we have f(a)=f(b) and g(b)<g(a).

If this still leaves several alternatives which are equally good, then we can optimize something else, and thus, have an even more complex optimality criterion.

In general, having an optimality criterion means that we are able to compare pairs of alternatives—at least some such pairs—and conclude that:

- for some of these pairs, we have a<b,
- for some of these pairs, we have b<a, and
- for some others pairs, we conclude that alternatives *a* and *b* are, from our viewpoint, of equal value; we will denote this by a∼b.

Of course, these relations must satisfy some reasonable properties. For example, if *b* is better than *a*, and *c* is better than *b*, then *c* should be better than *a*; in mathematical terms, the relation < must be transitive.

We must have some alternative which is better than or equivalent to all others—otherwise, the optimization problem has no solutions. It also makes sense to require that there is only one such optimal alternative—indeed, as we have mentioned, if there are several equally good optimal alternatives, this means that the original optimality criterion is not final, that we can use this non-uniqueness to optimize something else, i.e., in effect, to modify the original criterion into a final (or at least “more final”) one.

### 2.9. Invariance

There is an additional natural requirement for possible optimality criteria, which is related to the fact that the original “grid” Z×Z has lots of *symmetries*, i.e., transformations that transform this “grid” into itself.

For example, if we change the starting point of the coordinate system to a new point $\left(x_{0},y_{0}\right)$, then a point that originally had coordinates (x,y) now has coordinates $\left(x-x_{0},y-y_{0}\right)$. It makes sense to require that the relative quality of two different families F and $F^{\prime }$ will not change if we simply change the starting point.

Similarly, we can change the direction of the *x*-axis, then a point (x,y) becomes (−x,y). If we change the direction of the *y*-axis, we obtain a transformation (x,y)→(x,−y). Finally, we can rename the coordinates: what was *x* will become *y* and vice versa; this corresponds to the transformation (x,y)→(y,x). Such transformations should also not affect the relative quality of different families.

Please note that we are not requiring that the *family* F of sets be shift-invariant, what we require is that the *optimality criterion* is shift-invariant.

Let us explain why, in our opinion, it makes sense to require that the optimality criterion is shift-invariance—as well as having other invariance properties. Indeed, let us consider any usual optimality criterion such as accuracy of classification, robustness to noise, etc. What each criterion means is, e.g., the overall classification accuracy over the set S of all possible cat and not-a-cat images I∈S. We want this method to correctly classify images into cats and not-cats, whether these images are centered or somewhat shifted. Thus, to adequately compare different methods, we should test these methods on a set S of images that includes both original and shifted images.

Here:

- if we shift each image *I* from the set S by the same shift $\left(x_{0},y_{0}\right)$, i.e., replace each image I∈S by a shifted image $I^{\prime }=T_{x_{0},y_{0}}\left(I\right)$ for which $I^{\prime }\left(x,y\right)=I\left(x-x_{0},y-y_{0}\right)$,
- then, we should get, in effect, the exact same set of images:
  (24) $$T_{x_{0},y_{0}}\left(S\right)\overset{\mathrm{def}}{=}\left\{T\left(x_{0},y_{0}\right)\left(I\right):I\in S\right\}\approx S.$$

The only difference between these two sets of images may be the few images where the cat is right at the image’s boundary; in this paper, we will ignore this difference—just like we ignored the bounded-ness in the previous text. In this ignoring-bounds approximation, we conclude that

(25) $$T_{x_{0},y_{0}}\left(S\right)=\left\{T\left(x_{0},y_{0}\right)\left(I\right):I\in S\right\}=S.$$

How does a shift of the original image affect the input signals to the following convolution layers? In between the very first input layer and the following convolution layers, we may have (and usually do have) layers that perform the “compression” of the (x,y) part—i.e., that transform:

- values corresponding to several points (x,y)
- into values corresponding to a single new point $\left(x^{\prime },y^{\prime }\right)$.

In general, the (x,y)-shift of the original data corresponds to a shift of the transformed data—but by smaller shift values. For example, if data corresponding to each new (x,y)-point come from data from four different “pre-compression” points, then the shift by $\left(x_{0},y_{0}\right)$ in the pre-(x,y)-compression layer corresponds to a shift of the convolution layer input by $\left(x_{0}/2,y_{0}/2\right)$.

Since the set of input images should not change if we apply a shift, we can conclude that for each convolution layer, the set of the corresponding inputs to this layer should also not change if we shift all these inputs, i.e., if we replace each input $F_{d}\left(x,y\right)$ with a shifted input

(26) $$F_{d}^{\prime }\left(x,y\right)\overset{\mathrm{def}}{=}F_{d}\left(x-x_{0},y-y_{0}\right)$$

for some shift $\left(x_{0},y_{0}\right)$.

The set of inputs on which we compare different methods does not change when we apply a shift. So, if one method was better when we processed original inputs, it should still be better if we process shifted inputs—since the resulting set of inputs is the same. In other words, the quality (e.g., accuracy) $Q_{F}\left(S\right)$ of a method corresponding to the family F, when gauged by the set of inputs corresponding to original images, should be the same as this method’s quality $Q_{F}\left(T_{x_{0},y_{0}}\left(S\right)\right)$ on the set

(27) $$T_{x_{0},y_{0}}\left(S\right)=\left\{T_{x_{0},y_{0}}\left(F_{d}\right):F_{d}\in S\right\}$$

of all the inputs obtained from the original set S by this shift—since these two sets of inputs are, in effect, the same set: $T_{x_{0},y_{0}}\left(S\right)=S$. Thus, $Q_{F}\left(T_{x_{0},y_{0}}\left(S\right)\right)=Q_{F}\left(S\right)$.

However, as one can see, shifting all the inputs is equivalent to shifting all the sets from the family F. Indeed, if we apply the Formula (23) to the shifted layer’s input $F_{d}^{\prime }\left(x,y\right)\overset{\mathrm{def}}{=}F_{d}\left(x-x_{0},y-y_{0}\right)$, we get

(28) $$G_{d}\left(x,y\right)=\;\sum_{d^{\prime }=1}^{d_{\mathrm{in}}}\left(\sum_{\left(x^{\prime },y^{\prime }\right)\in S_{F}\left(x,y\right):\;d_{\infty }\left(\left(x,y\right),\left(x^{\prime },y^{\prime }\right)\right)\leq L}K_{d}\left(x,x^{\prime },y,y^{\prime },d^{\prime }\right)\cdot F_{d^{\prime }}\left(x^{\prime }-x_{0},y^{\prime }-y_{0}\right)\right),$$

i.e., in terms of the shifted coordinates $X\overset{\mathrm{def}}{=}x-x_{0}$ and $Y\overset{\mathrm{def}}{=}y-x_{0}$ for which $x=X+x_{0}$ and $y=Y+y_{0}$, we get—taking into account that the distance $d_{\infty }$ does not change with shift—that:

(29) $$G_{d}\left(X,Y\right)=\sum_{d^{\prime }=1}^{d_{\mathrm{in}}}\left(\sum_{C^{\prime }:\;d_{\infty }\left(\left(X,Y\right),\left(X^{\prime },Y^{\prime }\right)\right)\leq L}K_{d}^{\prime }\left(X,X^{\prime },Y,Y^{\prime },d^{\prime }\right)\cdot F_{d^{\prime }}\left(x^{\prime }-x_{0},y^{\prime }-y_{0}\right)\right),$$

where we denoted

(30) $$K_{d}^{\prime }\left(X,X^{\prime },Y,Y^{\prime },d^{\prime }\right)\overset{\mathrm{def}}{=}K_{d}\left(X+x_{0},X^{\prime }+x_{0},Y+y_{0},Y^{\prime }+y_{0}\right),\;$$

and where $C^{\prime }$ denotes the condition $\left(X^{\prime }+x_{0},Y^{\prime }+y_{0}\right)\in S_{F}\left(X+x_{0},Y+y_{0}\right)$.

In terms of the family F, the main difference between the Formulas (23) and (29) is that instead of the condition $\left(x^{\prime },y^{\prime }\right)\in S_{F}\left(x,y\right)$, we now have a new condition

(31) $$C^{\prime }\Leftrightarrow \left(X^{\prime }+x_{0},Y^{\prime }+y_{0}\right)\in S_{F}\left(X+x_{0},Y+y_{0}\right),\;$$

i.e., equivalently, $\left(X^{\prime },Y^{\prime }\right)\in S_{F}\left(X+x_{0},Y+y_{0}\right)-\left(x_{0},y_{0}\right)$. It is easy to check that this new condition is equivalent to $\left(Y^{\prime },Y^{\prime }\right)\in S_{F^{\prime }}\left(X,Y\right)$, where the new family $F^{\prime }$ is obtained by shifting sets from the original family F.

So:

- the relative quality of two families does not change if we shift all the layer’s inputs;
- however, shifting all the layer’s inputs is equivalent to shifting all the sets from the family F.

Thus, the relative quality of two families does not change if we shift both families. In other words, a reasonable optimality criterion—which describes which family is better—should be invariant with respect to shifts.

Similarly, we can argue that a reasonable optimality criterion should not change if we rename *x*- and *y*-axes, etc.

Now, we are ready for the precise formulation of the problem.

## 3. Definitions and the Main Result

**Definition** **1.** *By a* family*, we mean a family of non-empty subsets of the “grid” Z×Z, a family in which:*

- *all sets from this family are disjoint, and*
- *at least one set from this family has more than one element.*

*Terminological comment.* To avoid possible misunderstandings, let us emphasize that here, we consider several levels of sets, and to avoid confusion, we use different terms for sets from different levels:

- first, we consider *points* (x,y)∈Z×Z;
- second, we consider *sets* of points S⊆Z×Z; we call them simply sets;
- third, we consider sets of sets of points $F=\{S,S^{\prime },\ldots \}$; we call them *families*;
- finally, we consider the set of all possible families $\left\{F,F^{\prime },\ldots \right\}$; we call this a *class*.

*Comment about the requirements.* In the previous text, we argued that for each family F, the union of all its sets ∪{S:S∈F} should coincide with the whole “grid” Z×Z. However, in our definition of an alternative, we did not impose this requirement. We omitted this requirement to make our result stronger—since, as we see from the following Proposition, this requirement actually follows from all the other requirements.

**Definition** **2.** *By an* optimality criterion*, we mean a pair of relations (<,∼) on the class of all possible families that satisfy the following conditions:*

- *if $F<F^{\prime }$ and $F^{\prime }<F^{\prime }$, then $F<F^{\prime \prime }$;*
- *if $F<F^{\prime }$ and $F^{\prime }∼F^{\prime \prime }$, then $F<F^{\prime \prime }$;*
- *if $F∼F^{\prime }$ and $F^{\prime }<F^{\prime \prime }$, then $F<F^{\prime \prime }$;*
- *if $F∼F^{\prime }$ and $F^{\prime }∼F^{\prime \prime }$, then $F^{\prime }∼F^{\prime \prime }$;*
- *we have F∼F for all F; and*
- *if $F<F^{\prime }$, then we cannot have $F∼F^{\prime }$.*

*Comment.* The pair of relations (<,∼) between families of subsets forms what is called a *pre-order* or *quasi-order*. This notion is more general than partial order, since, in contrast to the definition of the partial order, we do not require that if a≤b and b≤a, then a=b: in principle, we can have a∼b for some a≠b.

**Definition** **3.** *We say that a family F is* optimal *with respect to the optimality criterion (<,∼), if for every other family $F^{\prime }$, we have either $F^{\prime }<F$ or $F^{\prime }∼F$.*

**Definition** **4.** *We say that the optimality criterion is* final *if there exists exactly one family which is optimal with respect to this criterion.*

**Definition** **5.** *By a* transformation *T:Z×Z, we mean one of the following transformations: $T_{x_{0},y_{0}}\left(x,y\right)=\left(x-x_{0},y-y_{0}\right)$, $T_{-+}\left(x,y\right)=\left(-x,y\right)$, $T_{+-}\left(x,y\right)=\left(x,-y\right)$, and $T_{↔}\left(x,y\right)=\left(y,x\right)$.*

**Definition** **6.** *For each family F and for each transformation T, by the* result T(F)*of applying the transformation T to the family F, we mean the family T(F)={T(S):S∈F}, where, for any set S, $T\left(S\right)\overset{\mathrm{def}}{=}\left\{T\left(x,y\right):\left(x,y\right)\in S\right\}.$*

**Definition** **7.** *We say that the optimality criterion is* invariant *if for all transformations T, $F<F^{\prime }$ implies that $T\left(F\right)<T\left(F^{\prime }\right)$, and $F∼F^{\prime }$ implies that $T\left(F\right)∼T\left(F^{\prime }\right)$.*

**Proposition** **1.** *For every final invariant optimality criterion, the optimal family is equal, for some integer ℓ≥1, to one of the following two families:*

- *the family of all the sets $S_{\ell ,x_{0},y_{0}}\overset{\mathrm{def}}{=}\left\{\left(x_{0}+\ell \cdot n_{x},y_{0}+\ell \cdot n_{y}\right):n_{x},n_{y}\in Z\right\}$ corresponding to all possible pairs of integers $\left(x_{0},y_{0}\right)$ for which $0\leq x_{0},y_{0}<\ell$;*
- *the family of all the sets*
  $$S_{\ell ,x_{0},y_{0}}^{\prime }\overset{\mathrm{def}}{=}\left\{\left(x_{0}+\ell \cdot n_{x},y_{0}+\ell \cdot n_{y}\right):n_{x},n_{y}\in Z\;and\;n_{x}+n_{y}\;is\;even\right\}$$
*corresponding to all possible pairs of integers $\left(x_{0},y_{0}\right)$ for which $0\leq x_{0},y_{0}<\ell$.*

*Comments.*

**Figure 7:**
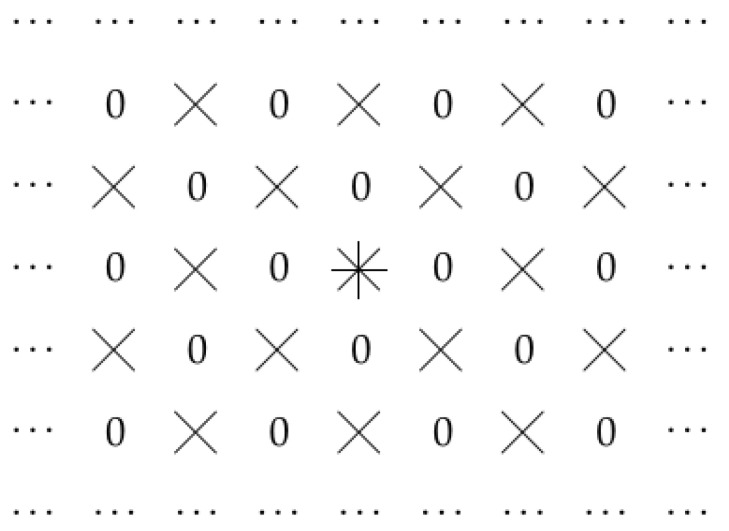
A set from the second possibly optimal family.

- This proposition takes care of all invariant (and final) optimality criteria. Thus, it should work for all usual criteria based on misclassification rate, time of calculation, used memory, or any others used in neural networks: indeed, if one method is better than another for images in general, it should remain to be better if we simply shift all the images or turn all the images upside down. Images can come as they are, they can come upside down, they can come shifted, etc. If, for some averaging criterion, one method works better for all possible images, but another method works better for all upside-down versions of these images—which is, in effect, the same class of possible images—then, from the common sense viewpoint, this would mean that something is not right with this criterion.
- The first possibly optimal case corresponds to dilated convolution. In the second possibly optimal case, the optimal family contains similar but somewhat different sets; an example of such a set is given in Figure 7.
  Thus, this result explains the effectiveness of dilated convolution—and also provides us with a new alternative worth trying.
- The following proof is similar to several proofs presented in [5,6].

**Proof.**  $1^{\circ }$. Since the optimality criterion is final, there exists exactly one optimal family $F_{\mathrm{opt}}$. Let us first prove that this family is itself invariant, i.e., that $T\left(F_{\mathrm{opt}}\right)=F_{\mathrm{opt}}$ for all transformations *T*.

Indeed, the fact that the family $F_{\mathrm{opt}}$ is optimal means that for every family F, we have $F<F_{\mathrm{opt}}$ or $F∼F_{\mathrm{opt}}$. Since this is true for every family F, it is also true for every family $T^{-1}\left(F\right)$, where $T^{-1}$ denotes inverse transformation (i.e., a transformation for which $T(T^{-1}\left(x,y\right))=\left(x,y\right)$). Thus, for every family F, we have either $T^{-1}\left(F\right)<F_{\mathrm{opt}}$ or $T^{-1}\left(F\right)∼F_{\mathrm{opt}}$. Due to invariance, we have $F=T\left(T^{-1}\left(F\right)\right)<T\left(F_{\mathrm{opt}}\right)$ or $F∼T(F_{\mathrm{opt}})$. By definition of optimality, this means that the alternative $T(F_{\mathrm{opt}})$ is also optimal. However, since the optimality criterion is final, there exists exactly one optimal family, so $T\left(F_{\mathrm{opt}}\right)=F_{\mathrm{opt}}$.

The statement is proven.

$2^{\circ }$. Let us now prove that the optimal family contains a set $S^{\prime }$ that, in its turn, contains the point (0,0) and some point (x,y)≠(0,0).

Indeed, by definition of a family, every family—including the optimal family—contains at least one set *S* that has at least two points. Let *S* be any such set from the optimal family, and let $\left(x_{0},y_{0}\right)$ be any of its points. Then, due to Part 1 of this proof, the set $S^{\prime }\overset{\mathrm{def}}{=}T_{x_{0},y_{0}}\left(S\right)$ also belongs to the optimal family, and this set contains the point

$$T_{x_{0},y_{0}}\left(x_{0},y_{0}\right)=\left(x_{0}-x_{0},y_{0}-y_{0}\right)=\left(0,0\right).$$

Since the set *S* had at least two different points, the set $S^{\prime }=T_{x_{0},y_{0}}\left(S\right)$ also contains at least two different points. Thus, the set $S^{\prime }$ must contain a point (x,y) which is different from (0,0).

The statement is proven.

$3^{\circ }$. In the following text, by $S^{\prime }$, we will mean a set from the optimal family $F_{\mathrm{opt}}$ whose existence is proven in Part 2 of this proof: namely, a set that contains the point (0,0) and a point (x,y)≠(0,0).

$4^{\circ }$. Let us prove that if the set $S^{\prime }$ contains a point (x,y), then this set also contains the points (x,−y), (−x,y), and (y,x).

Indeed, due to Part 1 of this proof, with the set $S^{\prime }$ the optimal family $F_{\mathrm{opt}}$ also contains the set $T_{+-}\left(S^{\prime }\right)$. This set contains the point $T_{+-}\left(0,0\right)=\left(0,0\right)$. Thus, the sets $S^{\prime }$ and $T_{+-}\left(S^{\prime }\right)$ have a common element (0,0). Since different sets from the optimal family must be disjoint, it follows that the sets $S^{\prime }$ and $T_{+-}\left(S^{\prime }\right)$ must coincide. The set $T_{+-}\left(S^{\prime }\right)$ contains the points (x,−y) for each point (x,y)∈S. Since $T_{+-}\left(S^{\prime }\right)=S^{\prime }$, this implies that for each point $\left(x,y\right)\in S^{\prime }$, we have $\left(x,-y\right)\in T_{+-}\left(S^{\prime }\right)=S^{\prime }$.

Similarly, we can prove that $\left(-x,y\right)\in S^{\prime }$ and $\left(y,x\right)\in S^{\prime }$. The statement is proven.

$5^{\circ }$. By combining the two conclusions of Part 4—that $\left(x,-y\right)\in S^{\prime }$ and that therefore $T_{-+}\left(x,-y\right)=\left(-x,-y\right)\in S^{\prime }$, we conclude that for every point $\left(x,y\right)\in S^{\prime }$, the point

$$-\left(x,y\right)\overset{\mathrm{def}}{=}\left(-x,-y\right)$$

is also contained in the set $S^{\prime }$.

$6^{\circ }$. Let us prove that if the set $S^{\prime }$ contains two points $\left(x_{1},y_{1}\right)$ and $\left(x_{2},y_{2}\right)$, then it also contains the point

$$\left(x_{1},y_{1}\right)+\left(x_{2},y_{2}\right)\overset{\mathrm{def}}{=}\left(x_{1}+x_{2},y_{1}+y_{2}\right).$$

Indeed, due to Part 1 of this proof, the set $T_{-x_{2},-y_{2}}\left(S^{\prime }\right)$ also belongs to the optimal family. This set shares an element

$$T_{-x_{2},-y_{2}}\left(0,0\right)=\left(0-\left(-x_{2}\right),0-\left(-y_{2}\right)\right)=\left(x_{2},y_{2}\right)$$

with the original set $S^{\prime }$. Thus, the set $T_{-x_{2},-y_{2}}\left(S^{\prime }\right)$ must coincide with the set $S^{\prime }$. Due to the fact that $\left(x_{1},y_{1}\right)\in S^{\prime }$, the element

$$T_{-x_{2},-y_{2}}\left(x_{1},y_{1}\right)=\left(x_{1}-\left(-x_{2}\right),y_{1}-\left(-y_{2}\right)\right)=\left(x_{1}+x_{2},y_{1}+y_{2}\right)$$

belongs to the set $T_{x_{1},y_{1}}\left(S^{\prime }\right)=S^{\prime }$. The statement is proven.

$7^{\circ }$. Let us prove that if the set $S^{\prime }$ contains a point (x,y), then, for each integer *c*, this set also contains the point

c·(x,y)=(c·x,c·y).

Indeed, if *c* is positive, this follows from the fact that

(c·x,c·y)=(x,y)+…+(x,y)(ctimes).

When *c* is negative, then we first use Part 5 and conclude that $\left(-x,-y\right)\in S^{\prime }$, and then conclude that the point (|c|·(−x),|c|·(−y))=(c·x,c·y) is in the set $S^{\prime }$.

$8^{\circ }$. Let us prove that if the set $S^{\prime }$ contains points $\left(x_{1},y_{1}\right)$, …, $\left(x_{n},y_{n}\right)$, then for all integers $c_{1},\ldots ,c_{n}$, it also contains their linear combination

$$c_{1}\cdot \left(x_{1},y_{1}\right)+\ldots +c_{n}\cdot \left(x_{n},y_{n}\right)=\left(c_{1}\cdot x_{1}+\ldots +c_{n}\cdot x_{n},c_{1}\cdot y_{1}+\ldots +c_{n}\cdot y_{n}\right).$$

Indeed, this follows from Parts 6 and 7.

$9^{\circ }$. The set $S^{\prime }$ contains some points which are different from (0,0), i.e., points for which at least one of the integer coordinates is non-zero. According to Parts 4 and 5, we can change the signs of both *x* and *y* coordinates and still get points from $S^{\prime }$. Thus, we can always consider points with non-negative coordinates.

Let *d* denote the greatest common divisor of all positive values of the coordinates of points from $S^{\prime }$.

If a value *x* appears as an *x*-coordinate of some point $\left(x,y\right)\in S^{\prime }$, then, due to Part 4, we have $\left(x,-y\right)\in S^{\prime }$ and thus, due to Part 5,

$$\left(x,y\right)+\left(x,-y\right)=\left(2x,0\right)\in S^{\prime }.$$

Similarly, if a value *y* appears as a *y*-coordinate of some point $\left(x,y\right)\in S^{\prime }$, then we get $\left(0,2y\right)\in S^{\prime }$ and thus, due to Part 3, $\left(2y,0\right)\in S^{\prime }$.

It is known that a common divisor *d* of the values $v_{1},\ldots ,v_{n}$ can be represented as a linear combination of these values:

$$d=c_{1}\cdot v_{1}+\ldots +c_{n}\cdot v_{n}.$$

For each value $v_{i}$, we have $\left(2v_{i},0\right)\in S^{\prime }$, thus, for

$$2d=c_{1}\cdot \left(2v_{1}\right)+\ldots +c_{n}\cdot \left(2v_{n}\right),$$

by Part 8, we get $\left(2d,0\right)\in S^{\prime }$. Due to Part 4, we thus have $\left(0,2d\right)\in S^{\prime }$, and due to Parts 6 and 7, all points $\left(n_{x}\cdot \left(2d\right),n_{y}\cdot \left(2d\right)\right)$ for integers $n_{x}$ and $n_{y}$ also belong to the set $S^{\prime }$.

If $S^{\prime }$ has no other points, then for the set containing (0,0), we indeed conclude that this set is the same as what we described for dilated convolution, with ℓ=2d.

$10^{\circ }$. What if there are other points in the set $S^{\prime }$? Since *d* is the greatest common divisor of all the coordinate values, each of these points has the form $\left(c_{x}\cdot d,c_{y}\cdot d\right)$ for some integers $c_{x}$ and $c_{y}$. Since this point is not of the form $\left(n_{x}\cdot \left(2d\right),n_{y}\cdot \left(2d\right)\right)$, this means that either $c_{x}$, or $c_{y}$ is an odd number—or both.

Let us first consider the case when exactly one of the values $c_{x}$ and $c_{y}$ is odd. Without losing generality, let us assume that $c_{x}$ is odd, so $c_{x}=2n_{x}+1$ and $c_{y}=2n_{y}$ for some integers $n_{x}$ and $n_{y}$. Due to Part 9, we have $\left(2n_{x}\cdot d,2n_{y}\cdot d\right)\in S^{\prime }$, so the difference

$$\left(\left(2n_{x}+1\right)\cdot d,2n_{y}\cdot d\right)-\left(2n_{x}\cdot d,2n_{y}\cdot d\right)=\left(d,0\right)$$

also belongs to the set $S^{\prime }$. Thus, similarly to Part 9, we can conclude that for every two integers $c_{x}$ and $c_{y}$, we have $\left(c_{x}\cdot d,c_{y}\cdot d\right)\in S^{\prime }$. So, in this case, $S^{\prime }$ coincides, for ℓ=d, with the set corresponding to dilated convolution.

The only remaining case is when not all points $\left(c_{x}\cdot d,c_{y}\cdot d\right)$ belong to the set $S^{\prime }$. This means that for some such point, both values $c_{x}$ and $c_{y}$ are odd: $c_{x}=2n_{x}+1$ and $c_{y}=2n_{y}+1$ for some integers $n_{x}$ and $n_{y}$. Due to Part 9, we have $\left(2n_{x}\cdot d,2n_{y}\cdot d\right)\in S^{\prime }$, so the difference

$$\left(\left(2n_{x}+1\right)\cdot d,\left(2n_{y}+1\right)\cdot d\right)-\left(2n_{x}\cdot d,2n_{y}\cdot d\right)=\left(d,d\right)$$

also belongs to the set $S^{\prime }$.

Since, due to Part 9, we have $\left(2n_{x}\cdot d,2n_{y}\cdot d\right)\in S^{\prime }$ for all $n_{x}$ and $n_{y}$, we conclude, by using Part 5, that $\left(\left(2n_{x}+1\right)\cdot d,\left(2n_{y}+1\right)\cdot d\right)\in S^{\prime }$. So, all pairs for which both coordinates are odd multiples of *d* are in $S^{\prime }$. Thus, we get the new case described in the Proposition.

$11^{\circ }$. The previous results were about the sets containing the point (0,0).

For all other sets *S* containing some other point $\left(x_{0},y_{0}\right)$, we get the same result if we take into account that the optimal family is invariant, and thus, with the set *S*, the optimal family also contains the set $T_{x_{0},y_{0}}\left(S\right)$ that contains (0,0) and is, thus, equal either to the family corresponding to dilated convolution or to the new similar family.

The proposition is proven. □

## 4. Conclusions and Future Work

### 4.1. Conclusions

One of the efficient machine learning ideas is the idea of a convolutional neural network. Such networks use convolutional layers, in which the layer’s output at each point is a combination of the layer’s input corresponding to several neighboring points. A reasonable idea is to restrict ourselves to only some of the neighboring points. It turns out that out of all such restrictions, the best results are obtained when we only use neighboring points, for which the differences in both coordinates are divisible by some constant *ℓ*. Networks implementing such restrictions are known as dilated convolutional neural networks.

In this paper, we provide a theoretical explanation for this empirical conclusion.

### 4.2. Future Work

This paper describes a general abstract result: that for any optimality criterion that satisfies some reasonable properties, some dilated convolution is optimal. To be practically useful, it is desirable to analyze which dilated convolutions are optimal for different practical situations and for specific criteria uses in machine learning, such as misclassification rate, time of calculation, used memory, etc. (and the combination of these criteria). It is also desirable to analyze what size neighborhood we should choose for different practical situations and for different criteria.

## Data Availability

Not applicable.
